# Key Information Influencing Patient Decision-Making About AI in Health Care: Survey Experiment Study

**DOI:** 10.2196/75615

**Published:** 2026-01-12

**Authors:** Xuan Zhu, Austin M Stroud, Sarah A Minteer, Dong Whi Yoo, Jennifer L Ridgeway, Maryam Mooghali, Jennifer E Miller, Barbara A Barry

**Affiliations:** 1 Robert D and Patricia E Kern Center for the Science of Health Care Delivery, Mayo Clinic, 200 First Street SW, Rochetser, MN, 55901, USA, 1 507-266-7916; 2 Biomedical Ethics Research Program, Mayo Clinic, Rochester, MN, USA; 3 Department of Physical Medicine and Rehabilitation Research, Mayo Clinic, Rochester, MN, USA; 4 Human-Centered Computing Department, Luddy School of Informatics, Computing, and Engineering, Indiana University, Indianapolis, IN, USA; 5 Division of Health Care Delivery Research, Mayo Clinic, Rochester, MN, USA; 6 Department of Internal Medicine, Yale School of Medicine, New Haven, CT, USA

**Keywords:** artificial intelligence, health communication, health decision-making, patient preference, patient-centered care, AI labeling

## Abstract

**Background:**

Artificial intelligence (AI)–enabled devices are increasingly used in health care. However, there has been limited research on patients’ informational preferences, including which elements of AI device labeling enhance patient understanding, trust, and acceptance. Clear and effective patient-facing communication is essential to address patient concerns and support informed decision-making regarding AI-enabled care.

**Objective:**

We evaluated 3 aims using simulated AI device labels in a cardiovascular context. First, we identified key information elements that influence patient trust and acceptance of an AI device. Second, we examined how these effects varied based on patient characteristics. Third, we explored how patients evaluated informational content of AI labels and their perceived effectiveness of the AI labels in informing decision-making about the use of AI device, building trust in the device, and shaping their intention to use it in their health care.

**Methods:**

We recruited 340 US patients from ResearchMatch.org to participate in a web-based survey that contained 2 experiments. In the discrete choice experiment, participants indicated preferences in terms of trust and acceptance regarding 16 pairs of simulated AI device labels that varied across 8 types of information needs identified in our previous qualitative work. In the single profile factorial experiment, participants evaluated 4 randomly assigned label prototypes regarding the label’s legibility, comprehensibility, information overload, credibility, and perceived effectiveness in informing about the AI device, as well as participants’ trust in the AI device and intention to use the device in their health care. Data were analyzed using mixed effects binary or ordinal logistic regression.

**Results:**

The discrete choice experiment showed that information about regulatory approval, high device performance, provider oversight, and AI’s value added to usual care significantly increased the likelihood of patient trust by 14.1%‐19.3% and acceptance by 13.3%‐17.9%. Subgroup analyses revealed variations based on patient characteristics such as familiarity with AI, health literacy, and recency of last medical checkup. The single profile factorial experiment showed that patients reported good label comprehension, and that information about provider oversight, regulatory approval, device performance, and AI’s added value improved perceived credibility and effectiveness of the AI label (odds ratio [OR] range: 1.35‐2.05), reduced doubts in the AI device (OR range: 0.61‐0.77), and increased trust and intention to use the AI device (OR range: 1.47‐1.73). However, information about data privacy and safety management protocols was less influential.

**Conclusions:**

Patients value information about an AI device’s performance, provider oversight, regulatory status, and added value during decision-making. Providing transparent, easily understandable information about these aspects is critical to support patient determinations of trust and acceptance of AI-enabled health care. Information elements’ impact on patient trust and acceptance varies by patient characteristics, highlighting the need for a tailored approach to address the concerns of diverse patient groups about AI in health care.

## Introduction

Artificial intelligence (AI) and machine learning (ML) are increasingly being integrated into health care products and services due to their potential to enhance diagnostic accuracy, improve treatment planning, increase efficiency of health care systems, reduce costs, and ultimately improve patient outcomes [1-3]. Cardiology, in particular, has seen a surge in AI/ML-enabled clinical tools, aiding clinical decision-making across the care spectrum [4-6]. While there are many promising applications of AI/ML in health care, effective approaches to inform patients about these technologies have not been established. This hinders informed patient decision-making and public trust in AI/ML-enabled medical devices.

Recent research on patient perspectives regarding AI in health care indicates that patients are generally receptive to the use of AI in their health care, but that certain conditions must be met [7,8]. Patients want clear and accessible information about how AI is used in their care, its benefits and limitations, how decisions are made, and the roles of AI and health care providers in AI-informed decisions [9-11]. Patients value human oversight over AI and want to ensure that health care decisions are ultimately made by a human [12-14]. Patients also want assurance that their data are protected and used responsibly [15-17]. Finally, they want AI to be unbiased in its training data and outputs and desire equitable access to AI applications to prevent against underrepresentation of minoritized and disadvantaged population groups in AI research [18-20]. These nuances underscore the complexity of information needed to support informed patient use of AI and effective communication between patients, clinicians, and health systems regarding the use of AI in care.

Recent efforts to understand how to communicate transparently about AI in health care have focused on clinical and technical audiences. As a result, there is growing research on best practices for transparent and standardized documentation, reporting, and communication of AI/ML models for clinicians [21-25]. However, research efforts focusing on identifying and prioritizing patients’ specific information needs remain limited [26-28]. It is important to note that it is not always feasible or desirable to present all information about an AI-enabled medical device with equal prominence in patient-facing communication. In practice, patient education materials, including labels, decision aids, and informed consents, must balance completeness with cognitive load. Overwhelming patients with too much information can reduce comprehension, informed decision-making, and psychological well-being [29-32]. Similarly, patient-facing labels for AI-enabled medical devices are highly constrained by space and by the cognitive burden on users [33-35]. Thoughtful organization and prioritization of information are key to avoiding information overload and making it easier for patients to grasp the implications of AI in their care [36]. Thus, to ensure patient safety, autonomy, and informed decision-making, it is essential to understand patients’ core information needs and present information in a transparent and accessible manner.

This study is part of a broader research project aimed at bridging the knowledge gap in effective communication about AI in health care by identifying the essential components of patient-facing information labels for these technologies. Information labels are structured summaries that provide users with key details about a device or technology, including its purpose, functionality, benefits, limitations, and risks [37]. The overarching goal of this project is to provide empirical evidence to inform strategies and regulatory policies that facilitate patient-centered adoption of AI in health care. For the purposes of our study, an AI device refers to a cardiology device equipped with an ML model designed to assist in decision-making across the continuum of care. Prior to this study, we conducted qualitative research, including a rapid literature review and 3 qualitative studies with patients and clinicians, to identify core information needs that contribute to patient and clinician trust in AI devices in health care, focusing on their use in diagnosing, treating, and monitoring cardiovascular conditions [34,38,39]. This work identified eight elements reported by patients as influential to their trust in the AI device, including (1) data privacy and security, (2) performance, (3) AI’s added value compared with usual care, (4) regulatory approval, (5) expert endorsement, (6) generalizability and limitations, (7) device safety, and (8) health care provider (HCP) oversight. However, no evidence-based guidance currently exists on how to prioritize among them in patient-facing communication. Understanding what information patients prioritize allows AI developers, health care systems, and regulators to emphasize the most critical information elements while ensuring transparency, clarity, and comprehension. To address this knowledge gap, this study used 2 survey experiments in which participants evaluated a short, hypothetical scenario (“vignette”) featuring AI label prototypes to achieve the following specific aims: (1) determine the relative importance of various information elements on patient trust and acceptance of an AI device in cardiology; (2) examine how the effects of information elements vary due to differences in patient characteristics, including familiarity with AI, medical mistrust, health literacy, and sociodemographic characteristics; and (3) assess patients’ evaluation of the informational content of the AI label prototypes, perceived effectiveness of the AI label prototypes in informing decision-making about the use of the AI device, trust in the device, and intention to use the device in their health care.

## Methods

### Clinical Context for Experimental Vignettes

We selected a hypothetical AI-enabled smartwatch and smartphone app designed to detect potential episodes of atrial fibrillation as the AI device example for our experimental vignettes. This choice was informed by input from our clinical and regulatory collaborators as well as feedback from both clinicians and patients during our formative qualitative research. The clinical problem and AI technology in the vignette were deemed appropriate by clinician collaborators given its direct relevance to patients and accessibility to a broad population. In addition, during formative qualitative research, this AI device prompted rich discussion among patient and clinician participants, underscoring its relevance and making it a strong candidate for use as the clinical context for the experimental vignettes.

### Experimental Design

#### Overview

To robustly examine how AI label informational content influences patient perceptions and decision-making, we conducted 2 vignette experiments through a web-based survey. The use of vignette experiments is a widely accepted approach in health communication and medical decision science research [40-43]. This method is especially useful when studying emerging technologies for which standardized communication practices are not yet established. The vignette experiment approach offers important advantages over simply asking participants to rank or rate the importance of various information elements [41]. By presenting information elements in concrete vignettes, we create a context that more closely resembles how decisions are made in real-world health care settings. It also allowed us to control for confounding factors and precisely isolate the causal effect of each information element on psychological and behavioral outcomes such as trust and acceptance. Moreover, simply asking participants what is important to them is highly susceptible to social desirability bias; by examining how participants respond to concrete situations, the vignette experiment approach minimizes social desirability bias and offers a clearer picture of the factors that shape patient preferences and decisions. To capture both patient priorities under conditions requiring trade-offs and their evaluations of AI label informational content in a more reflective context, we implemented 2 complementary vignette experiments: a discrete choice experiment (DCE) and a single profile factorial experiment (SPFE).

DCEs are being increasingly used in medical and health services research to systematically examine people’s preferences regarding health care services by assessing how much they value the specific attributes of the service [44-47]. In a typical DCE, participants are presented with 2 or more discrete hypothetical alternatives (eg, treatment A or treatment B), each consisting of multiple attributes with varying values. By analyzing the choices participants make among these alternatives, researchers can estimate the contribution of each attribute to decision-making. In this study, by observing how participants make trade-offs when choosing between competing label configurations, the DCE allowed us to (1) determine the relative importance of different information elements influencing patient trust and acceptance of an AI device and (2) examine how the effects of information elements vary due to differences in patient characteristics. This method uncovers underlying priorities and preferences that might not be evident when participants simply rate or rank information elements individually.

In contrast, the SPFE asked participants to review AI label prototypes one at a time and respond to a series of rating measures on perceived comprehension, cognitive effort, effectiveness in supporting decision-making about the AI device, trust in the device, and intention to use it in their health care. This method was chosen for 2 key reasons. First, the single profile design mirrors real-world patient decision-making regarding AI device use in health care more closely as in real life, patients typically consider a single device rather than make direct comparisons among alternatives. Second, the SPFE allows rating-based outcome measures (eg, 1=very unlikely to accept to 5=very likely to accept) that provide more direct and fine-grained insight into participants’ evaluation of the label prototypes as well as the psychological and behavioral impacts of the information elements. This dual-experiment approach allows us to gain a richer and more nuanced understanding of patient preferences regarding AI device labeling and the factors that are critical for effective communication and adoption of AI technologies in health care.

For these experiments, we created AI label prototypes based on the hypothetical AI device that varied on the following eight informational elements: (1) data privacy and security, (2) performance, (3) AI’s added value, (4) regulatory approval, (5) expert endorsement, (6) generalizability and limitations, (7) device safety, and (8) HCP oversight. We varied the informational content along these 8 elements based on prior qualitative research conducted with clinicians and patients, which identified these as important information needs influencing patient trust in AI [38,39]. For experimental design efficiency, each of these elements has 2 levels (ie, 2 variations): either in 2 different versions or as either present or absent. See Table 1 for additional details about these 8 elements.

**Table 1.** Label information elements and levels.

Elements	Definitions	Levels and examples
Elements with varying levels		
X1. Data privacy and security	What patient data are collected by AI^a^; how the patient data are being collected, stored, and shared.	Level 1: opt-in data sharing; level 2: opt-out data sharing
X2. Performance	True-positive: device accuracy when the patient DOES have A-fib^b^; true-negative: device accuracy when the patient DOES NOT have A-fib	Level 1: high performance; level 2: low performance
X3. AI’s added value	Improvement in patient care due to the AI-enabled device; effectiveness of the AI-enabled device in comparison with non–AI-enabled tests or conventional health care.	Level 1: information absent; level 2: information present
X4. Regulatory approval	Whether the AI device received clearance, approval, or certification from a regulatory body regarding its safety and/or effectiveness.	Level 1: information absent; level 2: information present
X5. Expert endorsement	Endorsement of the device for safety and effectiveness issued by medical experts such as health care providers.	Level 1: information absent; level 2: information present
X6. Generalizability and limitations	How generalizable is the device to patients with varying demographics and characteristics; what are the conditions or contexts where the device should not be used?	Level 1: internal validation; level 2: external validation
X7. Device safety	What are the risks of malfunction, bugs, and errors from the AI device (both the AI algorithm and the supporting software)? How will these risks be managed?	Level 1: proactive auditing; level 2: reactive auditing
X8. HCP^c^ oversight	Whether the results or decisions from the AI-enabled device have been verified by your health care provider.	Level 1: information absent; level 2: information present
Elements to be displayed in the same way across all labels		
1. Purpose	What is the purpose of the AI device, for what types of patients and conditions, when and in what context should the device be used?	“This AI-enabled smartwatch and smartphone app is designed to identify a potential episode of atrial fibrillation (A-fib). A-fib is an irregular and often very rapid heart rhythm that can lead to blood clots in the heart.”
2. Directions	How to use the AI device, recommended actions for patients, how to interpret results (eg, what is “normal” for pt. like me); what warrants discussion with provider; next steps by AI if any (eg, AI will automatically alert HCP)	“- Wear your smartwatch - It will provide an alert when it detects a potential episode of A-fib - Talk to your doctor if you received an alert from the app”

a AI: artificial intelligence.

b A-fib: atrial fibrillation.

c HCP: health care provider.

#### Discrete Choice Experiment

**Figure 1.**
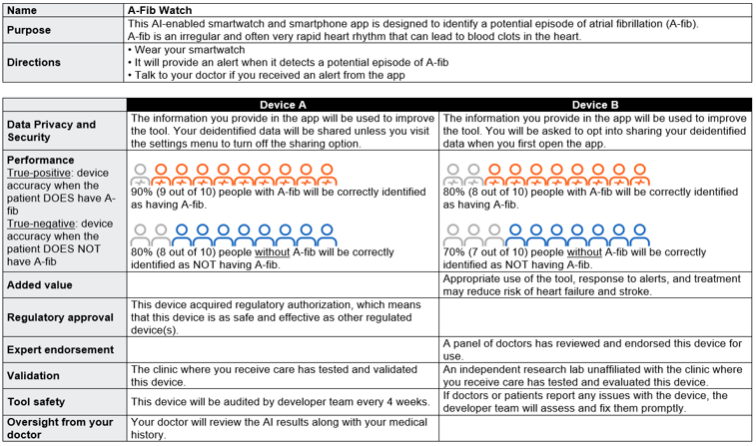
Example choice set for the discrete choice experiment. A-fib: atrial fibrillation; AI: artificial intelligence.

We used a fractional factorial design to rate a selection of possible alternatives because a full factorial design (2^8^ alternatives) would not be feasible to implement. To minimize the cognitive burden for participants and maximize statistical efficiency, each choice task consisted of 2 alternatives, and each participant was asked to consider 16 choice sets, comprising 32 unique label prototypes in total [48]. We used the R package idefix [49] to select an optimal design based on D-efficiency criterion, a commonly used metric for efficient experimental design construction to maximize statistical efficiency and precision [50-52]. Multimedia Appendix 1 summarizes the choice sets. Figure 1 shows an example choice set.

#### Single Profile Factorial Experiment

**Figure 2.**
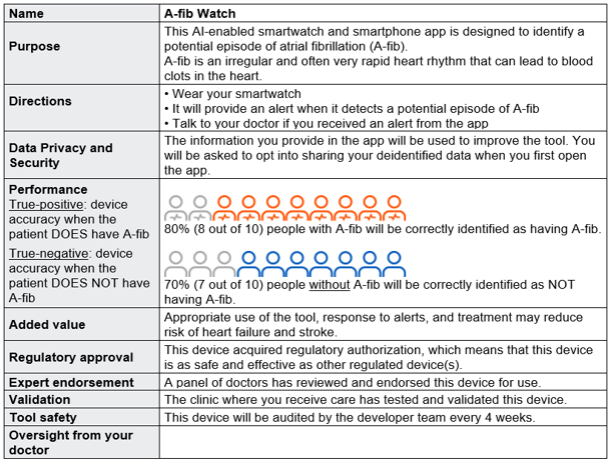
Example label prototype for the single profile factorial experiment. AI: artificial intelligence.

The SPFE used a 2^8-3^ resolution IV fractional factorial design to evaluate the label prototypes. We used the R package FrF2 [53] to generate the 32 experimental conditions (Multimedia Appendix 2). Each participant was randomly assigned to evaluate 4 label prototypes displayed in a randomized order. After viewing each label prototype, participants answered a series of questions on label evaluation, AI trust, and behavioral intention using 5-point Likert-style scales. Figure 2 shows an example label prototype.

### Pilot Testing

We conducted cognitive interviews (via Zoom [Zoom Communications, Inc]; 30‐45 minutes in length) with 4 participants to assess whether the information elements, variations, presentation, and survey questions were easy to understand and meaningful to participants. The interviewer shared the web-based survey with participants via shared screen and asked participants to verbally answer the survey questions and share their thought processes. The interviewer also asked participants to explain the information elements in their own words. The interviews were audio-recorded, and the interviewer took notes on reflections and observations. We revised the survey questionnaire based on interview notes.

Following the cognitive interviews, we pilot tested the revised Qualtrics web-based survey with 30 participants to examine the feasibility and data quality of the 2 vignette experiments. The vignette experiments consisted of 3 sections: DCE, SPFE, and participant characteristics. The presentation order of the 2 experiments was randomized to mitigate order effect. For the DCE, participants first completed a warm-up task to familiarize them with the DCE procedure. They were then given the following instructions for the main DCE on AI information labeling:

*Next, you will be asked to choose between 2 AI-enabled device options with similar or different characteristics.*

*AI-enabled devices in health care are medical devices that use AI and specifically the subset of AI known as machine learning. Some examples of AI-enabled devices in health care include smartwatches that can monitor your heart rate for problems, smart robots that guide surgeries, or AI programs that can provide information to a physician to help with diagnosis, among many other types of technologies.*

*Imagine that your health care provider recommended you using an AI-enabled smartwatch or smartphone app to help track your cardiac functions and identify potential episodes of atrial fibrillation. You were given 2 options. The 2 options were developed by the same company and provide the same functions. Please consider the 2 options below and choose the device you prefer the most*.

Participants then proceeded to complete 18 choice tasks, including 16 experimental choice tasks and 2 validity check tasks. The order of the 16 experimental choice tasks was randomized.

**Figure 3.**
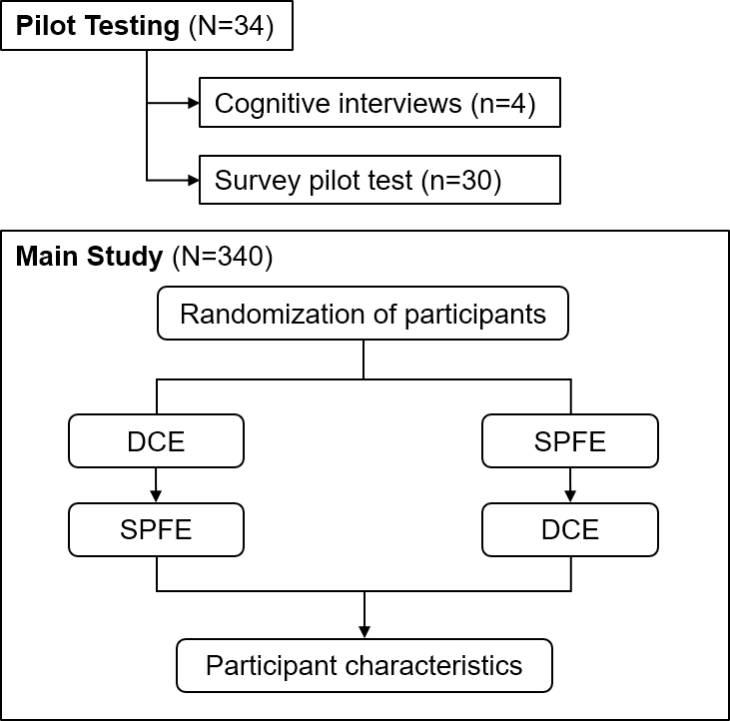
Study procedure. DCE: discrete choice experiment: SPFE: single profile factorial experiment.

In the SPFE, participants were randomly assigned to evaluate 4 of 32 label prototypes, presented in a random order. They were given the following instructions at the beginning of the section, “In this section, you will see 4 information labels about AI-enabled devices one at a time. After reading each information label, you will be asked a series of questions about your opinions on the label.” For each label prototype, participants answered questions on label evaluation, trust in the AI device, and intention to use the device in health care. After completing the experiments, participants provided information on their sociodemographic and health care characteristics. Figure 3 summarizes study procedure.

### Main Study

For the main study, participants completed the finalized web-based Qualtrics survey, which incorporated refinements from the pilot phase. The survey experiments followed the same procedures described in the “Pilot Testing” section, and the randomization procedure and data collection methods and materials remained consistent with the piloted version.

#### Measures

Label preference was measured through participants selecting their preferred label prototype in a choice task based on trust (“Which device would you trust more?”) and acceptance (“If you were given the option, which device would you be more likely to use in your health care?”).

Label evaluation included six items on a 5-point Likert scale (1=strongly disagree to 5=strongly agree) that assessed (1) legibility (“This label is easy to read”), (2) comprehensibility (“This label is easy to understand”), (3) information overload (“Reading this label is too mentally demanding for me”), (4) credibility (“I trust the information on this label”), and (5) effectiveness of the label in informing patients about the AI device (“This label gives me all the information I need about the AI device” and “This label helps me decide whether the AI device should be used in my care”).

Participants’ trust in the AI device was measured with three items on a 5-point Likert scale (1=strongly disagree to 5=strongly agree), including (1) “I would trust the results from this AI device,” (2) “I would have doubts about this AI device,” and (3) “I would seek a second opinion.” Participants’ likelihood of using the device was assessed on a 5-point Likert-type scale (1=very unlikely to 5=very likely). In addition, participants provided feedback on each label prototype through an optional open-ended question.

Participant characteristics were measured in terms of the need for cognition, which reflects motivation to process complex information and engage in effortful thinking [54,55], familiarity with AI, medical mistrust [56], health literacy [57-59], last routine medical checkup, health insurance coverage, and demographics (eg, age, gender, race or ethnicity, education level, and household financial status) [60].

#### Sample Size

We conducted an a priori power analysis to estimate the smallest sample size required for the main study. Because the DCE is more statistically efficient than the SPFE, we based our estimation on the SPFE design. The analysis showed that a sample of 288 participants would allow us to detect a main effect with a standardized regression coefficient of 0.167 or higher with 80% power at an α level of .05, which is sufficient for the DCE [61]. The total sample size needed for the pilot and main studies is 323. We planned to recruit 350 participants in total to account for attrition and incomplete responses.

#### Recruitment

We recruited participants from ResearchMatch [62], a national health volunteer registry that was created by several academic institutions and supported by the US National Institutes of Health as part of the Clinical Translational Science Award program. ResearchMatch has a large population of volunteers who have consented to be contacted by researchers about health studies for which they may be eligible. Inclusion criteria for this study include being an adult aged 18 years or older, being proficient in reading and writing in English, and having had a primary care or cardiology care visit within the past 3 years. We oversampled racial and ethnic minority patients to ensure that their representation was comparable with that of non-Hispanic White patients. This supported our ability to draw meaningful conclusions about diverse patient populations while accounting for historical underrepresentation in health research. Participants received a message describing our study via the ResearchMatch platform, where they could then decide to opt in to be contacted and receive a link to our survey. ResearchMatch provided contact information (ie, name and email address) for participants who opted in. Participants who opted in received an email invitation with a link to complete the online survey.

### Ethical Considerations

Review and approval for this study and all procedures were obtained from the Mayo Clinic Institutional Review Board (IRB; 21-012302). The IRB granted a waiver of written documentation of informed consent. The first page of the survey displayed an IRB-approved informed consent cover letter which provided information about the study, including its purpose, the investigator, the estimated length of the survey, data storage procedures, and contact information for the study team and the Mayo Clinic IRB. Participants were instructed to review this information and were informed that by proceeding to the survey, they were providing informed consent. No personal information was collected or stored with the survey responses. Study data were secured on institutionally approved and controlled access electronic storage. Participants received a US $10 gift card as remuneration for completing the survey.

### Statistical Analysis

We used mixed effects binary logistic regression to analyze the data from the DCE. Our analysis focused on how different information factors influenced the probability of individuals trusting and accepting the AI device in their health care. We included individual-specific intercepts to account for heterogeneity in individual preferences. We reported average marginal effect (AME), which shows the average change in predicted probabilities (percentage point increase or decrease) of the outcome variable across all participants when moving from one level of the information factor to another, while holding all other variables constant. In addition, we conducted subgroup analyses to explore how the effect of top preferred information factors on patient trust and acceptance of the AI device varied based on participants’ AI familiarity, openness toward medical AI, health literacy, medical mistrust, and sociodemographic characteristics.

We used mixed effects ordinal logistic regression (cumulative link mixed model) to analyze the data from the SPFE. Our focus was on how different information factors influenced participants’ evaluation of the AI label’s legibility, comprehensibility, information overload, information credibility, perceived effectiveness in informing about the AI device, trust in the AI device, and intention to use it in health care. We included individual-specific intercepts to account for repeated measurements within individuals. For models on AI device trust and intention to use, we adjusted for participants’ evaluation of the AI label’s legibility, comprehensibility, information overload, information credibility, and perceived effectiveness in informing about the AI device. We used “flexible” thresholds in our models to allow the distance between the 4 cut points of the 5-point scales to vary freely. We reported odds ratios (ORs) for the effect of information factors.

All analyses were performed in R (version 4.3.1; R Core Team) [63]. “tidyverse” [64] was used for data wrangling and visualization, “lme4” [65] was used for fitting mixed effects binary logistic models, “ordinal” [66] was used for fitting mixed effects ordinal logistic models, and “marginaleffects” [67] was used for calculating AME.

### Reporting Guidelines

This study is reported in accordance with the CHERRIES (Checklist for Reporting Results of Internet E-Surveys) [68].

## Results

### Participant Characteristics

A total of 340 participants who completed at least 75% of the survey questions were included in the analysis. Table 2 summarizes participant characteristics. Participants were aged 18‐34 (122/328, 37.19%), 35‐54 (114/328, 34.75%), and 55 years or older (92/328, 28.05%). Nearly half were women (158/326, 48.47%), 48.77% (159/326) were men, and 2.76% (9/326) identifying as nonbinary or another gender. The majority were non-Hispanic White (206/327, 63.00%), followed by Black or African American (76/327, 23.24%), Hispanic or Latinx (44/327, 13.46%), Asian or Asian American (30/327, 9.17%), and Native American or Indigenous (13/327, 3.98%). Regarding education, 49.09% (161/328) had a bachelor’s degree, 28.96% (95/328) held a master’s degree or higher, and 20.73% (68/328) had some college or an associate degree. Financially, 53.54% (174/325) reported having disposable income after paying bills, 29.85% (97/325) had little spare money, and 16.62% (54/325) struggled to pay bills.

**Table 2.** Participant characteristics (N=340).

Characteristics	Values, n (%)
Age (years)	
18‐24	18 (5.49)
25‐34	104 (31.71)
35‐44	72 (21.95)
45‐54	42 (12.80)
55‐64	52 (15.85)
≥65	40 (12.20)
Missing	12 (3.52)
Gender	
Man	159 (48.77)
Woman	158 (48.47)
Nonbinary/other	9 (2.76)
Missing	14 (4.12)
Race and ethnicity^a^	
White/Caucasian	206 (63.00)
Black/African American	76 (23.24)
Hispanic/Latina/Latino	44 (13.46)
Asian/Asian American	30 (9.17)
Native American/American Indian/Alaska Native/Indigenous	13 (3.98)
Middle Eastern/North African/Arab American	8 (2.45)
Native Hawaiian/Pacific Islander	2 (0.61)
Other race	6 (1.83)
Missing	13 (3.82)
Education level	
High school or less	4 (1.22)
Some college or associate’s degree	68 (20.73)
Bachelor’s degree	161 (49.09)
Master’s degree or higher	95 (28.96)
Missing	12 (3.52)
Perceived household financial status	
After paying the bills, I still have enough money for special things that I want	174 (53.54)
Have enough money to pay the bills but little spare money to buy extra or special things	97 (29.85)
Have enough money to pay the bills but only because I have cut back on things	28 (8.62)
Having difficulty paying the bills, no matter what I do	26 (8.00)
Missing	15 (4.41)
Health insurance coverage	
Private	207 (65.51)
Public	103 (32.59)
No insurance	6 (1.90)
Missing	24 (7.06)
Last routine checkup	
Less than a year ago	212 (66.88)
>1 year, <2 years	53 (16.72)
>2 years, <5 years	41 (12.93)
≥5	6 (1.89)
Never had routine checkup	5 (1.58)
Missing	23 (6.76)

a The percentages may add over 100% because participants can select multiple races.

Most participants had private health insurance (207/316, 65.51%), while 32.59% (103/316) had public coverage. In addition, 66.88% (212/317) had a routine medical checkup within the past year.

### DCE Results

#### Importance of the Information Factors for Patients’ Trust in the AI Device

**Figure 4.**
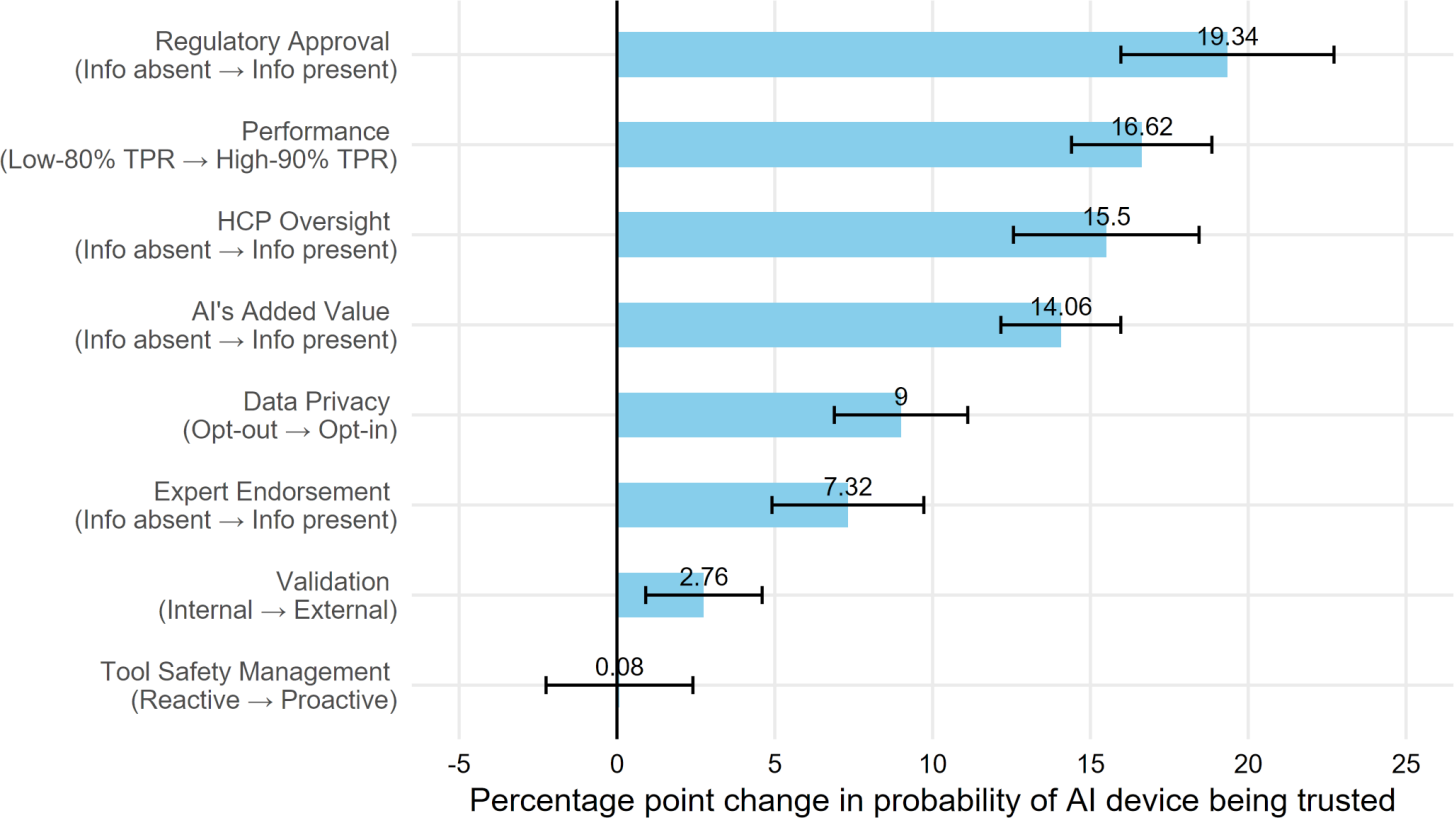
Difference in probability of AI device being trusted by attribute level. AI: artificial intelligence; HCP: health care provider; Info: information; TPR: true-positive rate.

Figure 4 visualizes the relative importance of the information factors for participants’ trust in the AI device. The 4 information factors that produced the greatest increase in participants’ trust in AI devices were inclusion of information about regulatory approval (AME=19.34%, 95% CI 15.97%-22.72%), information about high versus low performance (AME=16.62%, 95% CI 14.40%-18.85%), inclusion of information about HCP oversight (AME=15.50%, 95% CI 12.56%-18.44%), and inclusion of information about the AI’s added value compared with usual care (14.06% increase, 95% CI 12.16%-15.96%). Including information about opt-in (vs opt-out) data privacy protocol, expert endorsement (vs information absent), and information about external (vs internal) validation less strongly increased the probability of trusting the AI device (9%, 7.32%, and 2.76%, respectively). Effect of information about proactive versus reactive device safety management protocol on participants’ trust was not statistically significant (Table 3).

**Table 3.** Effects of information elements on the probability of the artificial intelligence device being trusted and accepted.

	Trust	Acceptance
	AME^a^ (95% CI), percentage points	AME^a^ (95% CI), percentage points
Added value (information absent→information present)	14.06 (12.16 to 15.96)	15.58 (13.28 to 17.88)
Data privacy (opt out→opt in)	9.00 (6.89 to 11.11)	10.92 (8.30 to 13.53)
Expert endorsement (information absent→information present)	7.32 (4.91 to 9.72)	9.03 (6.73 to 11.32)
HCP^b^ oversight (information absent→information present)	15.50 (12.56 to 18.44)	17.86 (14.98 to 20.75)
Performance (low→high)	16.62 (14.40 to 18.85)	14.85 (12.65 to 17.06)
Regulatory approval (information absent→information present)	19.34 (15.97 to 22.72)	13.29 (9.96 to 16.63)
Device safety (reactive→proactive)	0.08 (–2.25 to 2.41)	0.74 (–1.82 to 3.29)
Validation (internal→external)	2.76 (0.91 to 4.60)	3.64 (1.56 to 5.72)

a AME: average marginal effect. It is the average change in the predicted probabilities (percentage point increase or decrease) of the artificial intelligence device being trusted or accepted across all participants when moving from one level of the information factor to the other, keeping all other variables in the model constant.

b HCP: health care provider.

#### Importance of the Information Factors for Patients’ Acceptance of the AI Device

**Figure 5.**
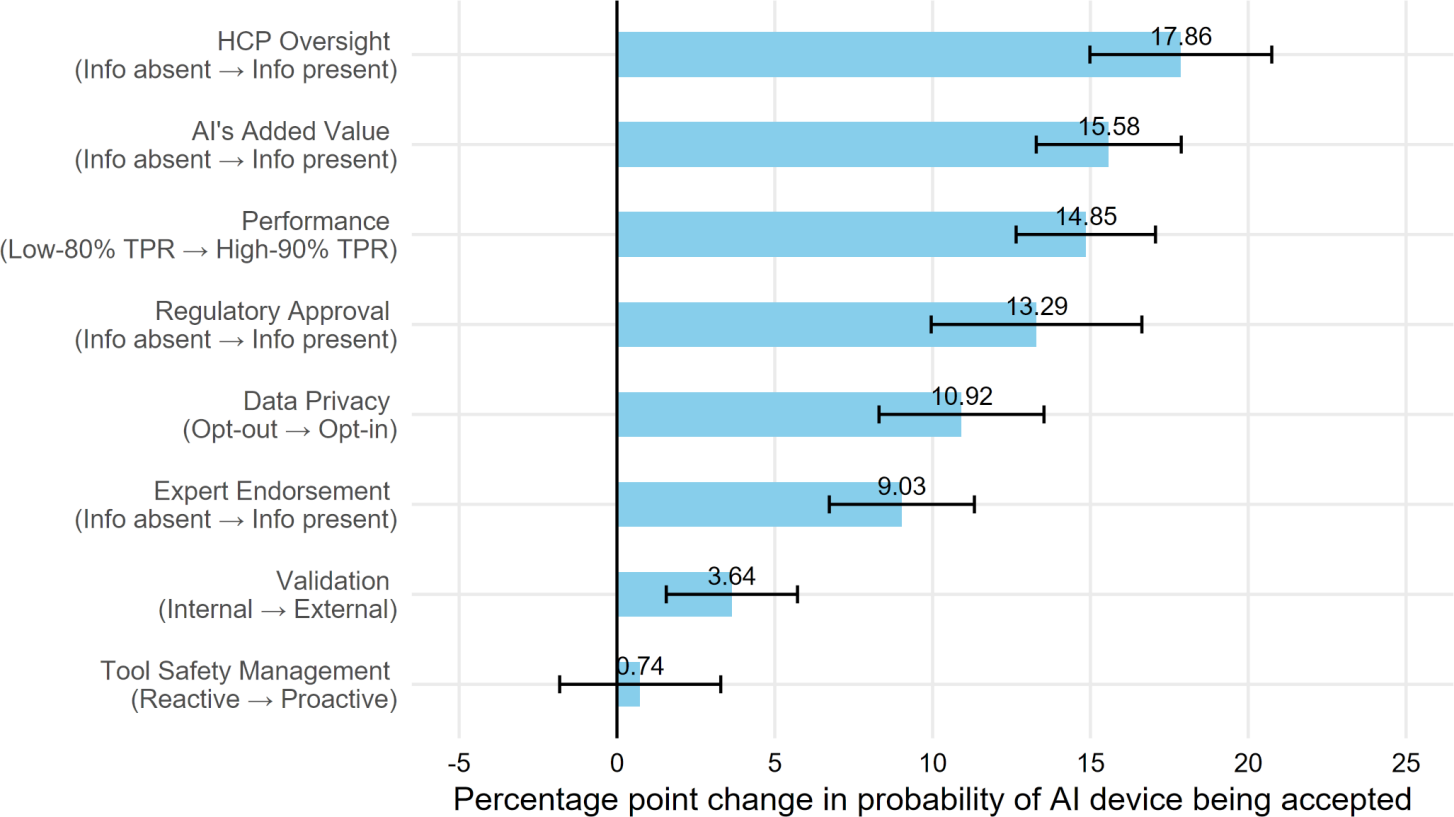
Difference in probability of artificial intelligence device being accepted by attribute level. AI: artificial intelligence; HCP: health care provider; Info: information; TPR: true-positive rate.

Figure 5 visualizes the relative importance of the information factors for participants’ willingness to use the AI device in their health care (ie, acceptance). The 4 information factors that produced the greatest increase in participants’ acceptance in using AI devices were inclusion of information about HCP oversight (17.86% increase, 95% CI 4.98%-20.75%), inclusion of information about the device’s added value (15.58% increase, 95% CI 13.28%-17.88%), information about high versus low device performance (14.85% increase, 95% CI 12.65%-17.06%), and inclusion of information about regulatory approval (13.29% increase, 95% CI 9.96%-16.63%). Including information about opt-in (vs opt-out) data privacy protocol, expert endorsement (vs information absent), and information about external (vs internal) validation less strongly increased the probability of the AI device being accepted (10.92%, 9.03%, and 3.64%, respectively). The effect of information about proactive versus reactive device safety management protocol on device acceptance was not statistically significant (Table 3).

#### Subgroup Differences in the Effect of Top Preferred Information Factors on Patient Trust and Acceptance

##### Subgroup Differences in Patient Trust

Our analyses revealed subgroup differences in how the top 4 most important information factors affected trust in AI devices, based on participants’ level of familiarity with AI, level of reading health literacy, level of need for cognition, recency of last routine medical checkup, age group, and gender (Multimedia Appendix 3).

###### AI’s Added Value

Specifically, having information about AI’s added value (vs information absent) had a stronger positive effect on participants’ trust in AI devices with a high (vs low) level of need for cognition (AME=16.21% vs 12.23%; *P*=.04).

###### HCP Oversight

Having information about HCP oversight (vs information absent) had a stronger positive effect on the probability of AI device being trusted for participants who reported not at all to somewhat (vs very or extremely) familiar with AI (AME=19.27% vs 11.30%; *P*=.01), for participants whose last routine checkup was less than a year ago (vs 1 or more years ago) (AME=18.27% vs 11.63%; *P*=.007), and for participants aged 55 years or older (vs aged 35‐54 or 18‐34 years) (AME=23.44% vs 10.34% or 15.25%; *P*<.001; *P*=.03).

###### High Device Performance

Information about high (vs low) device performance had a stronger positive effect on the probability of AI device being trusted for participants with a high (vs low) level of reading health literacy (AME=22.02% vs 11.06%; *P*<.001), for participants whose last routine checkup was less than a year ago (vs 1 or more years ago) (AME=20.09% vs 10.38%; *P*=.048), and for participants who identified as women (vs men) (AME=19.38% vs 12.85%; *P*=.005).

###### Regulatory Approval

Having information about regulatory approval (vs information absent) had a stronger positive effect on the probability of AI device being trusted for participants with a high (vs low) level of reading health literacy (AME=25.07% vs 13.63%; *P*=.001) and for participants whose last routine checkup was less than a year ago (vs 1 or more years ago) (AME=23.29% vs 12.92%; *P*<.001).

### Subgroup Differences in Patient Acceptance

There were subgroup differences in how the top 4 most important information factors affected acceptance of AI device, based on participants’ reported level of familiarity with AI, level of reading health literacy, level of numeracy, recency of last routine medical checkup, gender, and race or ethnicity (Multimedia Appendix 4).

#### AI’s Added Value

Specifically, having information about the device’s added value (vs information absent) had a stronger positive effect on the probability of the AI being accepted for participants who reported being not at all to somewhat familiar (vs very or extremely) with AI (AME=18.06% vs 11.49%; *P*=.005), for participants with a high (vs low) level of reading health literacy (AME=19.34% vs 11.96%; *P*=.002), for participants whose last routine checkup was within the last year (vs 1 or more years ago) (AME=19.19% vs 8.81%; *P*<.001), for participants who identified as women (vs men) (AME=18.94% vs 11.38%; *P*=.002), and for participants who identified as a person of color (vs non-Hispanic White) (AME=19.64% vs 12.01%; *P*=.001).

#### HCP Oversight

Providing information about HCP oversight (vs information absent) had a stronger positive effect on the probability of AI device being accepted for participants who reported not at all to somewhat familiar (vs very or extremely) with AI (AME=25.34% vs 9.59%; *P*<.001), for participants whose last routine checkup was within the last year (vs 1 or more years ago) (AME=21.92% vs 13.31%; *P*<.001), and for participants aged 55 years or older (vs aged 35‐54 or 18‐34 years) (AME=30.13% vs 13.05% or 13.77%; *P*<.001; *P*<.001).

#### High Device Performance

Information about high (vs low) device performance had a stronger positive effect on the probability of AI device being accepted for participants who reported not at all to somewhat familiar (vs very or extremely) with AI (AME=18.18% vs 10.58%; *P*<.001), for participants with a high (vs low) level of reading health literacy (AME=21.41% vs 7.66%; *P*<.001), for participants with a high (vs low) level of numeracy (AME=19.38% vs 9.77%; *P*<.001), for participants whose last routine checkup was within the last year (vs 1 or more years ago) (AME=18.91% vs 7.03%; *P*=.004), for participants who identified as women (vs men) (AME=18.71% vs 9.57%; *P*<.001), and for participants aged 55 years or older (vs aged 35‐54 or 18‐34 years) (AME=20.88% vs 12.80% or 11.82%; *P*=.006; *P*=.002).

#### Regulatory Approval

Providing information about regulatory approval (vs information absent) had a stronger positive effect on the probability of AI device being accepted for participants with a high (vs low) level of reading health literacy (AME=19.93% vs 5.82%; *P*<.001), for participants with a high (vs low) level of numeracy (AME=15.79% vs 9.16%; *P*=.004), for participants whose last routine checkup was within the last year (vs 1 or more years ago) (AME=18.65% vs 3.96%; *P*<.001), and for participants who identified as women (vs men) (AME=16.58% vs 9.43%; *P*=.04).

### SPFE Results

#### Descriptive Statistics

Table 4 summarizes the descriptive statistics of the AI label evaluation measures. Most participants reported that the AI labels were easy to read (perceived legibility; 86.7%) and understand (comprehensibility; 84.2%). They also reported that reading the labels was not too mentally demanding (no information overload; 63.9%). Effects of the information factors on perceived legibility, comprehensibility, and information overload were not statistically significant (Table 5).

**Table 4.** Descriptive statistics of the artificial intelligence label evaluation measures.

	5-point Likert scale, n (%)				
Measure	1	2	3	4	5
This label is easy to read (perceived legibility)^a^	15 (1.12)	67 (5.00)	96 (7.16)	474 (35.37)	688 (51.34)
This label is easy to understand (comprehensibility)^a^	18 (1.34)	73 (5.44)	121 (9.02)	498 (37.14)	631 (47.05)
Reading this label is too mentally demanding for me (information overload)^a^	524 (39.13)	332 (24.79)	125 (9.34)	202 (15.09)	156 (11.65)
I trust the information on this label (information credibility)^a^	30 (2.24)	94 (7.01)	220 (16.41)	597 (44.52)	400 (29.83)
Label gives all information needed about the AI^b^ device (perceived label effectiveness)^a^	44 (3.28)	243 (18.13)	177 (13.21)	502 (37.46)	374 (27.91)
Label helps me decide whether the device should be used in my care (perceived label effectiveness)^a^	15 (1.12)	83 (6.19)	162 (12.09)	600 (44.78)	480 (35.82)
I would trust the results from this AI device^a^	38 (2.83)	138 (10.29)	218 (16.26)	642 (47.87)	305 (22.74)
I would have doubts about this AI device^a^	128 (9.55)	351 (26.17)	274 (20.43)	405 (30.20)	183 (13.65)
I would seek a second opinion^a^	84 (6.26)	162 (12.08)	256 (19.09)	489 (36.47)	350 (26.10)
Use the AI device if offered the option^c^	22 (1.64)	125 (9.31)	229 (17.06)	521 (38.82)	445 (33.16)

a 1=strongly disagree; 2=somewhat disagree; 3=neither agree nor disagree; 4=somewhat agree; 5=strongly agree.

b AI: artificial intelligence.

c 1=very unlikely; 2=somewhat unlikely; 3=undecided; 4=somewhat likely; 5=very likely.

**Table 5.** Effects of information element on perceived legibility, comprehensibility, information overload, information credibility, and perceived effectiveness of the label in informing decision-making.

	Perceived legibility^b^	Comprehensibility^b^	Information overload^b^	Information credibility^b^	Perceived effectiveness of the label in informing decision-making^a^	
					Label gives all information needed about the AI^c^ device	Label helps me decide whether the device should be used in my care
	OR^d^ (95% CI)	OR (95% CI)	OR (95% CI)	OR (95% CI)	OR (95% CI)	OR (95% CI)
Added value						
Information present	0.94 (0.70 to 1.27)	1.17 (0.88 to 1.56)	0.79 (0.58 to 1.05)	1.35 (1.05 to 1.73)	1.36 (1.06 to 1.74)	1.15 (0.89 to 1.48)
Information absent	Reference	Reference	Reference	Reference	Reference	Reference
Data privacy						
Opt-in	1.11 (0.83 to 1.50)	1.07 (0.80 to 1.42)	0.84 (0.63 to 1.13)	1.09 (0.85 to 1.39)	0.71 (0.56 to 0.91)	0.97 (0.75 to 1.24)
Opt-out	Reference	Reference	Reference	Reference	Reference	Reference
Expert endorsement						
Information present	1.21 (0.90 to 1.63)	0.90 (0.68 to 1.21)	0.87 (0.65 to 1.16)	1.37 (1.07 to 1.76)	1.09 (0.85 to 1.39)	1.27 (0.98 to 1.63)
Information absent	Reference	Reference	Reference	Reference	Reference	Reference
HCP^e^ oversight						
Information present	1.11 (0.83 to 1.50)	1.13 (0.85 to 1.51)	0.88 (0.66 to 1.18)	1.60 (1.25 to 2.05)	1.48 (1.16 to 1.89)	1.41 (1.09 to 1.81)
Information absent	Reference	Reference	Reference	Reference	Reference	Reference
Performance						
High	1.21 (0.90 to 1.62)	1.15 (0.87 to 1.52)	0.84 (0.63 to 1.11)	1.45 (1.14 to 1.85)	1 (0.78 to 1.27)	1.16 (0.91 to 1.48)
Low	Reference	Reference	Reference	Reference	Reference	Reference
Regulatory approval						
Information present	1.16 (0.86 to 1.58)	1.29 (0.97 to 1.72)	0.99 (0.74 to 1.34)	1.55 (1.21 to 1.99)	2.05 (1.60 to 2.63)	1.11 (0.87 to 1.43)
Information absent	Reference	Reference	Reference	Reference	Reference	Reference
Device safety						
Proactive	0.92 (0.68 to 1.24)	0.91 (0.69 to 1.21)	1 (0.75 to 1.33)	1.03 (0.80 to 1.31)	0.90 (0.71 to 1.15)	1.09 (0.85 to 1.39)
Reactive	Reference	Reference	Reference	Reference	Reference	Reference
Validation						
External	0.98 (0.73 to 1.32)	1.13 (0.85 to 1.51)	1.01 (0.76 to 1.35)	1.01 (0.79 to 1.29)	1.14 (0.89 to 1.45)	1.03 (0.80 to 1.32)
Internal	Reference	Reference	Reference	Reference	Reference	Reference

a Model adjusted for perceived eligibility, comprehensibility, information overload, information credibility, and patient characteristics that were statistically significantly associated with the outcome.

b Model adjusted for patient characteristics that were statistically significantly associated with the outcome.

c AI: artificial intelligence.

d OR: odds ratio.

e HCP: health care provider.

#### Credibility

Having information about added value (OR 1.35, 95% CI 1.05-1.73), expert endorsement (OR 1.37, 95% CI 1.07-1.76), HCP oversight (OR 1.60, 95% CI 1.25-2.05), high (vs low) performance (OR 1.45, 95% CI 1.14-1.85), and regulatory approval (OR 1.55, 95% CI 1.21-1.99) were associated with higher levels of perceived credibility of the information (Table 5).

#### Perceived Effectiveness of Label

Information about added value (OR 1.36, 95% CI 1.06-1.74), HCP oversight (OR 1.48, 95% CI 1.16-1.89), and regulatory approval (OR 2.05, 95% CI 1.60-2.63) were associated with higher likelihood of participants reporting that the label gives them all the information they need about the AI device. Information about opt-in versus opt-out data privacy protocol (OR 0.71, 95% CI 0.56-0.91) was associated with lower likelihood of participants reporting that the label gives them all the information they need about the AI device. Information about HCP oversight (OR 1.41, 95% CI 1.09-1.81) was associated with higher likelihood of participants reporting that the AI label helps them decide whether the AI device should be used in their care (Table 5).

#### Trust and Intentions to Use AI

Information about expert endorsement (OR 1.30, 95% CI 1.01-1.68) and high (vs low) performance (OR 1.48, 95% CI 1.16-1.90) were associated with higher levels of trust in the results from the AI device. Information about high (vs low) performance (OR 0.77, 95% CI 0.61-0.98) and regulatory approval (OR 0.61, 95% CI 0.48-0.78) was associated with lower likelihood of participants reporting having doubts in the AI device. Information about HCP oversight (OR 0.75, 95% CI 0.57-0.97) and regulatory approval (OR 0.63, 95% CI 0.48-0.83) were associated with lower likelihood of participants reporting needing a second opinion. Information about HCP oversight (OR 1.47, 95% CI 1.12-1.94), high (vs low) performance (OR 1.59, 95% CI 1.22-2.07), and regulatory approval (OR 1.73, 95% CI 1.31-2.30) were associated with higher intention to use the AI device if offered the option (Table 6).

**Table 6.** Effects of information element on patient trust in the artificial intelligence device and intention to use the artificial intelligence device.

	Trust in the AI device^a^, OR^b^ (95% CI)			Intention to use the AI device if offered the option^a^, OR (95% CI)
	I would trust results from this AI^c^ device	I would have doubts about this AI device	I would seek a second opinion	
Added value				
Information present	0.83 (0.64 to 1.06)	1.15 (0.90 to 1.47)	0.87 (0.67 to 1.14)	0.99 (0.75 to 1.31)
Information absent	Reference	Reference	Reference	Reference
Data privacy				
Opt-in	1.07 (0.83 to 1.37)	1.07 (0.85 to 1.36)	1.27 (0.98 to 1.65)	1.03 (0.79 to 1.34)
Opt-out	Reference	Reference	Reference	Reference
Expert endorsement				
Information present	1.30 (1.01 to 1.68)	0.89 (0.70 to 1.13)	0.93 (0.71 to 1.21)	1.17 (0.89 to 1.54)
Information absent	Reference	Reference	Reference	Reference
HCP^d^ oversight				
Information present	1.21 (0.94 to 1.55)	0.80 (0.63 to 1.03)	0.75 (0.57 to 0.97)	1.47 (1.12 to 1.94)
Information absent	Reference	Reference	Reference	Reference
Performance				
High	1.48 (1.16 to 1.90)	0.77 (0.61 to 0.98)	0.80 (0.62 to 1.04)	1.59 (1.22 to 2.07)
Low	Reference	Reference	Reference	Reference
Regulatory approval				
Information present	1.26 (0.98 to 1.64)	0.61 (0.48 to 0.78)	0.63 (0.48 to 0.83)	1.73 (1.31 to 2.30)
Information absent	Reference	Reference	Reference	Reference
Device safety				
Proactive	1.08 (0.84 to 1.38)	1.13 (0.89 to 1.43)	0.98 (0.76 to 1.27)	0.93 (0.71 to 1.21)
Reactive	Reference	Reference	Reference	Reference
Validation				
External	1.06 (0.83 to 1.36)	1.12 (0.88 to 1.42)	0.80 (0.61 to 1.03)	0.97 (0.74 to 1.27)
Internal	Reference	Reference	Reference	Reference

a Model adjusted for perceived eligibility, comprehensibility, information overload, information credibility, perceived effectiveness of the label in informing decision-making, and patient characteristics that were statistically significantly associated with the outcome.

b OR: odds ratio.

c AI: artificial intelligence.

d HCP: health care provider.

## Discussion

### Overview

To our knowledge, this is the first study to apply 2 experimental methods, DCE and SPFE, to elicit patient preferences for information about the use of AI devices in their health care. Our study provides important evidence and insights for health care and AI professionals and policy makers on the relative importance of various information factors to patient decision-making, trust, and acceptance regarding an AI device in cardiology and on subgroup differences in the impact of top preferred information factors. This work is a first step toward developing effective communication strategies about AI in health care that ensure transparency and accessibility to facilitate informed decision-making and patient-centered adoption of AI applications.

### Study Design Considerations

Before delving into our findings, we first address the use of the vignette experiment approach, the hypothetical AI device, and the nonclinical study sample. While vignette experiments are a well-established method in health communication and medical decision science research, they are not without limitations. In particular, the artificial nature of vignettes can raise concerns about ecological validity, as hypothetical scenarios may not fully capture the complexities of real-world decision-making. As a result, there is a risk that the study findings cannot be generalized directly to real-world settings [40-43].

However, in the context of our study, the use of the vignette experiments with a hypothetical AI device and a nonclinical study sample was both methodologically necessary and ethically appropriate. Methodologically, this approach allowed us to standardize and systematically manipulate the information elements of interest, while holding all other aspects of the scenario constant. This level of experimental control is often impossible to achieve with a real AI device due to the presence of a range of uncontrolled confounding influences on patient decision-making, including prior experiences, provider communication, and exposure to media or marketing. Importantly, the ecological validity of vignette experiments depends not on literal replication of real-world settings but on whether the vignettes activated the same psychological processes that occur during real-life decision-making [41]. To enhance the realism and credibility of our vignettes, we designed the hypothetical AI device scenario in consultation with clinical and regulatory collaborators and informed by formative qualitative research with both clinicians and patients. We also sought clinician review on the vignette drafts to ensure clinical accuracy and appropriateness and then further refined their clarity and relevance by piloting through cognitive interviews with patients. These steps strengthened both the internal and ecological validity of our study design and increased the applicability of our findings to real-world AI communication contexts.

Moreover, testing experimental manipulations involving actual patients making real-time medical decisions may raise ethical concerns, particularly when issues surrounding patient trust, understanding, and decision-making about AI in health care are still evolving [69]. Our experiment focuses on comparing different information factors presented for the same device within a consistent clinical scenario. Conducting an empirical study using an actual AI device while manipulating its label would involve providing false information, such as presenting different performance metrics for the same AI device to different patients, which would constitute deception and be unethical. This approach would undermine patient autonomy in decision-making and could erode trust in their clinicians and health care system [70]. To ensure that the hypothetical scenario was relevant to study participants and consistent with real-world decision-making, we recruited adults who reported recent health care experiences through either a primary care or cardiovascular care visit within the past 3 years. Importantly, our study goal was to understand general patient preferences and responses to AI labeling across a diverse sample, which is a necessary first step before testing specific AI implementations in particular clinical populations.

### Key Findings

Results from the 2 experiments offer complementary insights into the key factors shaping patients’ decision-making about use of an AI device in cardiology. Results from the SPFE showed that most participants found the AI label prototypes easy to read and understand and not mentally demanding. The effects of information factors on perceived legibility, comprehensibility, and information overload were not statistically significant, indicating that patient preferences were not due to a lack of understanding of these labels. Results from the DCE showed that information about provider oversight, regulatory approval, high device performance, and AI’s added value were the most influential in increasing patient trust in the AI device and their willingness to use the device in their health care. Patients placed less importance on information about opt-in versus opt-out data privacy protocol, expert endorsement, external versus internal validation protocol, and proactive versus reactive device safety management protocol.

Results from the SPFE reinforced these findings. First, information about AI’s added value, expert endorsement, HCP oversight, high performance, and regulatory approval was linked to higher perceived credibility of the AI label. In addition, information about added value, HCP oversight, and regulatory approval increased the likelihood that participants felt that they had all the necessary information about the device. Interestingly, information about HCP oversight was the only factor that significantly influenced participants’ perception of the label’s usefulness in deciding whether to use the device in their care. Information about expert endorsement, high performance, and regulatory approval was also associated with greater trust in the AI device’s results and reduced doubts about the device. Furthermore, information about HCP oversight and regulatory approval lowered the likelihood of participants seeking a second opinion. Finally, information about HCP oversight, high performance, and regulatory approval contributed to a higher intention to use the AI device if offered.

The DCE also showed that participant characteristics, including recency of last medical checkup, familiarity with AI, health literacy, numeracy, need for cognition, age, gender, and race or ethnicity, shaped how strongly information about AI’s added value, device performance, HCP oversight, and regulatory approval impacted trust and acceptance of the AI device. Information about AI’s added value had greater effects among participants who had more recent medical checkups, were less familiar with AI, had higher health literacy, had higher need for cognition, identified as women, or identified as people of color. Similarly, information about high device performance had stronger effects among those with more recent medical checkups, lower familiarity with AI, higher health literacy or numeracy, older age, or identified as women. Information about HCP oversight had stronger effects among participants with more recent medical checkups, lower familiarity with AI, or older age. Finally, the effects of information about regulatory approval were stronger among those with recent checkups, higher health literacy or numeracy, or women.

### Implications for Practice

Our findings have important implications for the implementation of AI in health care. To start, providing transparent and accessible information about HCP oversight, regulatory approval, device performance, and the device’s added value is critical for building patient trust and acceptance of AI technology [27]. These 4 information elements were the most influential in shaping patients’ willingness to use an AI device, highlighting the need for health care systems to clearly communicate them when introducing AI devices to patients. Health care systems could consider developing decision aids that allow patients to explore different aspects of a specific AI device in collaboration with their providers, helping them weigh the benefits and risks of using the device in their care [26]. This is especially important for patients with lower baseline trust or limited familiarity with AI.

Our findings also highlight the critical importance of information about endorsement and oversight from regulatory bodies and HCPs in boosting patients’ confidence and reducing doubts about the AI device’s effectiveness and safety, which can lead to higher trust in the device and lower likelihood of needing second opinions. This strong preference for human oversight and approval echoes literature on algorithm aversion, which shows that people are often reluctant to trust algorithmic decision-making systems even when they outperform humans [71,72]. This aversion is especially pronounced in decisions involving high uncertainty [73] and for tasks perceived as subjective [74]; however, it can be mitigated when there is room for human oversight and modification [75]. While algorithm aversion may initially make patients hesitant to trust AI, patient trust in their provider and the health system can lead to inflated expectations of AI’s positive impact on care and potentially result in overreliance when patients lack the expertise to judge when to rely on it. Transparency and patient engagement are therefore critical to calibrating trust appropriately [28,76]. Notably, information about HCP oversight was particularly influential in helping patients decide whether an AI device should be used in their care. This finding is consistent with previous research showing that patients are more receptive to AI technologies when AI supports, rather than replaces, the decisions of trusted human HCPs, underscoring the value of a “human in the loop” approach to AI implementation that provides patients a sense of accountability and assurance regarding the safety and effectiveness of these technologies [12-14,18,77,78]. To improve patient engagement and acceptance of AI devices, patient-facing communications should explicitly emphasize providers’ active role in reviewing, interpreting, and overriding AI outputs.

Three information elements, opt-in versus opt-out data privacy, external versus internal validation, and proactive versus reactive device safety management protocols, were found to be relatively less influential in shaping participants’ trust and decision-making regarding the AI device. This variation in the prioritization of information elements reflects how patients actively calibrate trust in AI technologies and advances beyond prior empirical work that was limited to identifying information relevant to patient trust [26,28,39,79]. Specifically, patients tend to anchor their trust in the AI device on signals that are personally meaningful from a layperson’s perspective, such as information about HCP oversight, regulatory approval, expert endorsement, and device performance, rather than the more abstract procedural indicators such as data privacy, validation, and safety management. While critical from a policy and ethical standpoint [38], these issues may be too abstract or poorly understood by a layperson to effectively translate into meaningful differences in care [80]. In addition, the terms used in the descriptions of these elements, including “deidentified data,” “tested and evaluated,” “fix issues promptly,” and “device will be audited,” may have signaled to participants that basic privacy, validity, and safety protections were in place, especially in the presence of a strong credibility cue [81]—the study instruction that their HCP recommended the AI device. As a result, many participants may have relied on heuristics to reassure themselves about privacy, validity, and safety and focused their mental effort on other aspects that are more directly related to the benefits and risks of using the device in their care [82,83]. These findings echo previous research showing that increasing transparency alone is insufficient for calibrated trust, and such information must also be accessible and meaningful to users [35,80,84]. Technical or abstract aspects of the AI device should be contextualized in terms of impact on the patient’s care and communicated in accessible and relatable ways, for example, through plain language summaries, visuals, and metaphors, ideally developed through an iterative process of co-design and testing with patients [85-88]. These findings also highlight a potential tension between what patients find most useful in decision-making and what is required by regulators or ethics guidelines. For example, although data privacy is a central focus in recent AI regulations [89], participants in our study placed relatively lower importance on it. This suggests that patients may not benefit from regulatory-required information in the same way as other stakeholders and may sometimes prefer information that is not routinely made available as part of regulatory clearance processes. To support appropriately calibrated trust of AI technologies in health care, patient-facing communication strategies should emphasize the information patients value most, while also clearly addressing critical ethical and regulatory considerations [90]. Importantly, to make the abstract concepts more relatable, ethical and regulatory features should be framed in terms of their tangible benefits and risks to patients. Communication efforts must also strike a careful balance: offering reassurance while avoiding overstating benefits or certainty, which could create misplaced confidence in AI technology [76,91].

Moreover, patient-facing communication and education about AI in health care should be tailored to meet the unique needs and preferences of different patient groups [92,93]. For example, information about a device’s added value was found to be particularly influential for patients who were less familiar with AI, had high reading health literacy, had recent routine checkups, and identified as women and people of color. Emphasizing the role of HCPs in overseeing AI use was found to be especially important for patients who are less familiar with AI, had recent routine checkups, and were aged 55 years or older. To effectively address these diverse patient needs and preferences, communication strategies could include tailoring language to patient reading levels and incorporating visual aids, infographics, and videos to help patients better understand complex information about the AI device and make informed decisions about their care. In addition, engaging patients, patient advocacy groups, and community organizations in co-designing patient-facing AI communication and educational materials including AI device labels could help address concerns from underrepresented groups who may have different levels of comfort with and trust in AI technologies and the health care system [94]. Providing AI communication and education materials in various formats and at different time points may improve the accessibility of information about AI and better meet the diverse needs of different patient groups. Health care systems can consider offering in-person consultations during clinical visits, along with printed and digital materials for patients to take home, ensuring that patients have the opportunity to review and understand the information at their own pace [39]. Moreover, it is the responsibility of health care systems to ensure that providers are well informed about AI devices and are capable of communicating their benefits and risks effectively to diverse patient populations. Health care systems could establish standardized guidelines and best practices for patient-facing communication and education about AI in health care. Training providers on how to effectively discuss AI devices with diverse patient populations and address their concerns would be instrumental in ensuring that patients receive clear, transparent information that aids informed decision-making and builds merited trust in AI technologies. Table 7 summarizes key findings and implications for practice.

**Table 7.** Summary of key findings, implications, and recommendations.

Findings	Implications and recommendations
High impact information Information about HCP^a^ oversight, regulatory approval, high device performance, and AI’s^b^ added value led to largest increases in trust and willingness to use. These elements also boosted the AI label’s credibility, increased trust in the results, reduced doubts, and enhanced patient confidence in having enough information about the device for decision-making.	Patients prioritize personally meaningful, concrete information over abstract procedural details. Clearly communicate information about HCP oversight, regulatory approval, device performance, and added value, as these elements are crucial for building patient trust and enabling acceptance of AI technologies. Develop decision aids that help patients and providers explore the benefits and risks of specific AI devices together, especially important for patients with lower baseline trust in or limited familiarity with AI.
Lower impact information: Participants placed less importance on information about opt-in versus opt-out data privacy, model validation protocol, and safety management protocol.	Abstract issues may be poorly understood, so patients rely on heuristics and focus on aspects directly related to benefits and risks. Technical or abstract aspects should be contextualized for patient care and communicated via plain language, visuals, and metaphors. Communication should emphasize what patients value while addressing critical ethical and regulatory considerations.
Role of human oversight: Information about HCP oversight and regulatory approval consistently boosted patient confidence, reduced doubts, increased trust, lowered the need for second opinions, and increased intention to use the AI-enabled device if offered.	Patients prefer AI that supports, rather than replaces, human decision makers, underscoring the importance of a “human in the loop” approach. Communicating provider oversight can inflate expectations of AI’s benefits; transparency and engagement are key to calibrating trust based on human oversight. Patient-facing communication should emphasize providers’ role in reviewing, interpreting, and approving or overriding AI outputs. Ensure that providers are well informed about AI devices and can effectively communicate benefits, risks, and limitations to patients.
Subgroup differences: Impact of information elements varied by participant characteristics. Added value: stronger effect for recent checkups, low AI familiarity, high literacy or need for cognition, women, and people of color. High performance: stronger for recent checkups, low AI familiarity, high literacy or numeracy, older age, and women. HCP oversight: stronger for recent checkups, low AI familiarity, and older age. Regulatory approval: stronger for recent checkups, high literacy or numeracy, and women.	Tailor language to patient reading levels and use visuals, infographics, and videos to explain complex AI information. Co-design materials with patients, advocacy groups, and community organizations to address diverse trust levels and concerns. Provide materials in multiple formats and at different time points to meet diverse patient needs. Establish standardized guidelines and best practices for patient-facing AI communication. Train providers on how to effectively discuss benefits, risks, and limitations of AI devices with diverse patient populations.

a HCP: health care provider.

b AI: artificial intelligence.

### Strengths, Limitations, and Future Directions

This research has several strengths. First, we used innovative experimental methods to elicit patient preferences for information about AI in health care, which could be applied to other use cases and medical specialties. Our methods can also be used to develop and evaluate other patient-facing AI communication and education materials. We applied a rigorous process for selection of information elements, including a rapid literature review and 3 qualitative studies, which strengthened the validity of our findings.

Our findings should be interpreted in the context of the limitations. The study sample, recruited from ResearchMatch.org, is a convenience sample and may not necessarily represent the US adult population. To address this, we intentionally oversampled racial and ethnic minority populations to ensure their representation and enable comparisons between people of color and non-Hispanic White patients in AI information preferences. Since nearly all participants had health insurance, caution is needed when generalizing the findings to uninsured populations. In addition, while selection bias might be a concern, we lack data to compare participants with nonparticipants, which may impact the generalizability of our findings. Nonetheless, our methods of evaluating the importance of information elements can be applied to other AI devices, medical conditions, and other types of AI communication and education efforts.

Our study sample may not closely reflect the demographic and clinical characteristics of patients most likely to use AI-enabled cardiology devices. While all study participants reported having had a primary care or cardiology visit within the past 3 years, we could not clinically verify that they were seeking cardiovascular care during the study period. Therefore, findings may not generalize to clinical populations actively facing decisions about AI-enabled cardiac care. Future research should replicate this work among clinical populations to validate and extend the findings. Furthermore, we used a hypothetical AI-enabled cardiology device to maintain experimental control and isolate the effects of specific information elements. However, this approach may not fully capture the complexity and nuance of real-world patient decision-making. As a result, the ecological validity of our findings may be limited. To build upon and extend the current findings, future research should examine how patients actively seeking cardiovascular care respond to labeling content for real-world AI-enabled devices in clinical practice.

Our study focused on the impact of patient-facing informational content in AI labeling, and it did not directly assess how provider recommendation might influence patient trust and acceptance of AI technologies. To hold provider recommendation constant across experimental conditions, we instructed all participants to assume that their provider had recommended the use of the hypothetical AI device. We acknowledge the important role of provider recommendation in patient adoption of AI technologies and encourage future research to explore how provider recommendation and label content interact to shape patient decision-making.

We examined patient information preferences and responses to AI label prototypes at a single time point prior to actual use of an AI device. As AI technologies become more integrated into routine health care and daily life, patients may gain greater familiarity with AI and shift information priorities after repeated exposure and use. Future research should adopt longitudinal study designs to examine how patient information needs, preferences, trust, and acceptance evolve over time and across different stages of their health care journey.

The information elements presented to participants were informed by our prior qualitative research; however, they may not fully reflect the complete range of information that could appear on an actual AI device label. In addition, because our study focused solely on the informational content of AI labels, the format, layout, and visual design of the label prototypes were simplified and standardized for ease of delivery through a web-based survey and may not fully reflect the look or feel of AI device labels used in real-world clinical practices. Future research is needed to examine how variations in display format (eg, static vs interactive), information modality (eg, textual vs infographic), delivery channel (eg, digital vs printed), and timing (eg, previsit, point-of-care, postvisit) influence patient comprehension, trust, and decision-making regarding AI technologies in health care. Finally, it is critical to involve patients in co-designing and testing AI communication strategies to ensure that the materials are accessible, engaging, and aligned with the needs and preferences of diverse patient populations.

### Conclusions

Through experimental studies, our research underscores the critical importance of transparent and accessible patient-facing information about AI devices and their impact on patient trust and acceptance. Information on HCP oversight, regulatory approval, device performance, and its added value emerged as pivotal factors influencing patient decision-making. Tailoring communication to meet the diverse needs and preferences of patient subgroups is essential for effective and equitable AI adoption in health care. Patient-centered communication strategies, coupled with comprehensive education for HCPs, would ensure that AI technologies are integrated into health care in a way that empowers patients to make informed choices and support overall patient care.

## Supplementary material

10.2196/75615 Multimedia Appendix 1 Choice sets.

10.2196/75615 Multimedia Appendix 2 2IV8-3 fractional factorial experimental design.

10.2196/75615 Multimedia Appendix 3 Subgroup differences in effects of information factors on the probability of the artificial intelligence device being trusted.

10.2196/75615 Multimedia Appendix 4 Subgroup differences in effects of information factors on the probability of the artificial intelligence device being accepted.

10.2196/75615 Checklist 1 CHERRIES checklist.

## Abbreviations

AI: artificial intelligence
AME: average marginal effect
CHERRIES: Checklist for Reporting Results of Internet E-Surveys
DCE: discrete choice experiment
HCP: health care provider
IRB: institutional review board
ML: machine learning
OR: odds ratio
SPFE: single profile factorial experiment
