# Supplementary material for: Key Information Influencing Patient Decision-Making About AI in Health Care: Survey Experiment Study
Source: J Med Internet Res. 2026 Jan 12;28:e75615. doi: 10.2196/75615 (PMC12795307; doi:10.2196/75615)
Supplement: Multimedia Appendix 1 [file jmir-v28-e75615-s001.docx]

Multimedia Appendix 1. Choice sets.

|  | Alternative A | | | | | | | | Alternative B | | | | | | | |
| --- | --- | --- | --- | --- | --- | --- | --- | --- | --- | --- | --- | --- | --- | --- | --- | --- |
|  | X1  Data privacy | X2 Performance | X3  Added value | X4  Regulatory approval | X5  Expert endorsement | X6  Validation | X7  Device safety | X8  HCP over  sight | X1  Data privacy | X2 Performance | X3  Added value | X4  Regulatory approval | X5  Expert endorsement | X6  Validation | X7  Device safety | X8  HCP over  sight |
| 1 | Opt-out | High | Off | Off | On | Internal | Reactive | On | Opt-out | High | Off | Off | On | External | Proactive | On |
| 2 | Opt-out | High | On | On | On | External | Reactive | On | Opt-out | High | Off | On | On | Internal | Proactive | Off |
| 3 | Opt-in | Low | On | Off | On | Internal | Reactive | On | Opt-out | High | On | On | Off | Internal | Reactive | Off |
| 4 | Opt-in | Low | Off | Off | Off | Internal | Proactive | On | Opt-out | Low | Off | On | On | Internal | Reactive | On |
| 5 | Opt-in | Low | Off | On | On | Internal | Reactive | Off | Opt-in | Low | Off | Off | Off | External | Reactive | Off |
| 6 | Opt-in | High | Off | On | On | Internal | Reactive | Off | Opt-out | Low | On | Off | Off | Internal | Proactive | On |
| 7 | Opt-in | High | Off | Off | Off | External | Reactive | On | Opt-in | Low | On | On | On | External | Reactive | Off |
| 8 | Opt-out | Low | On | On | On | External | Reactive | Off | Opt-out | High | Off | Off | On | External | Proactive | On |
| 9 | Opt-out | Low | Off | Off | Off | External | Proactive | On | Opt-out | High | Off | On | On | Internal | Proactive | Off |
| 10 | Opt-out | High | Off | Off | On | Internal | Reactive | On | Opt-out | High | On | On | Off | Internal | Reactive | Off |
| 11 | Opt-out | High | On | On | On | External | Reactive | On | Opt-out | Low | Off | On | On | Internal | Reactive | On |
| 12 | Opt-in | Low | On | Off | On | Internal | Reactive | On | Opt-in | Low | Off | Off | Off | External | Reactive | Off |
| 13 | Opt-in | Low | Off | Off | Off | Internal | Proactive | On | Opt-out | Low | On | Off | Off | Internal | Proactive | On |
| 14 | Opt-in | Low | Off | On | On | Internal | Reactive | Off | Opt-in | Low | On | On | On | External | Reactive | Off |
| 15 | Opt-in | High | Off | On | On | Internal | Reactive | Off | Opt-out | High | Off | Off | On | External | Proactive | On |
| 16 | Opt-in | High | Off | Off | Off | External | Reactive | On | Opt-out | High | Off | On | On | Internal | Proactive | Off |
