# Supplementary material for: Key Information Influencing Patient Decision-Making About AI in Health Care: Survey Experiment Study
Source: J Med Internet Res. 2026 Jan 12;28:e75615. doi: 10.2196/75615 (PMC12795307; doi:10.2196/75615)
Supplement: Multimedia Appendix 2 [file jmir-v28-e75615-s002.docx]

Multimedia Appendix 2. 2IV^8-3^ fractional factorial experimental design.

| Condition | X1. Data privacy and security | X2. Performance | X3. Added value | X4. Regulatory approval | X5. Expert endorsement | X6. Validation | X7. Device safety | X8. HCP oversight |
| --- | --- | --- | --- | --- | --- | --- | --- | --- |
| 1 | Opt-in | High | On | Off | Off | External | Reactive | On |
| 2 | Opt-in | High | On | Off | On | External | Proactive | Off |
| 3 | Opt-in | Low | On | Off | On | External | Proactive | On |
| 4 | Opt-out | Low | On | On | Off | External | Proactive | Off |
| 5 | Opt-in | Low | On | Off | Off | External | Reactive | Off |
| 6 | Opt-out | Low | Off | Off | On | External | Proactive | Off |
| 7 | Opt-out | Low | Off | Off | Off | External | Reactive | On |
| 8 | Opt-out | High | On | Off | Off | Internal | Proactive | Off |
| 9 | Opt-out | High | Off | On | On | Internal | Proactive | Off |
| 10 | Opt-out | Low | Off | On | Off | Internal | Reactive | Off |
| 11 | Opt-in | High | Off | On | On | External | Reactive | On |
| 12 | Opt-out | Low | Off | On | On | Internal | Proactive | On |
| 13 | Opt-in | High | Off | Off | On | Internal | Reactive | Off |
| 14 | Opt-in | Low | On | On | Off | Internal | Reactive | On |
| 15 | Opt-out | High | Off | On | Off | Internal | Reactive | On |
| 16 | Opt-in | Low | Off | Off | On | Internal | Reactive | On |
| 17 | Opt-out | Low | On | On | On | External | Reactive | On |
| 18 | Opt-in | Low | Off | Off | Off | Internal | Proactive | Off |
| 19 | Opt-out | Low | On | Off | On | Internal | Reactive | Off |
| 20 | Opt-in | High | On | On | On | Internal | Proactive | On |
| 21 | Opt-in | Low | On | On | On | Internal | Proactive | Off |
| 22 | Opt-out | High | On | On | On | External | Reactive | Off |
| 23 | Opt-out | High | On | On | Off | External | Proactive | On |
| 24 | Opt-in | Low | Off | On | Off | External | Proactive | On |
| 25 | Opt-out | High | Off | Off | On | External | Proactive | On |
| 26 | Opt-in | High | On | On | Off | Internal | Reactive | Off |
| 27 | Opt-in | High | Off | On | Off | External | Proactive | Off |
| 28 | Opt-in | Low | Off | On | On | External | Reactive | Off |
| 29 | Opt-out | High | Off | Off | Off | External | Reactive | Off |
| 30 | Opt-out | High | On | Off | On | Internal | Reactive | On |
| 31 | Opt-in | High | Off | Off | Off | Internal | Proactive | On |
| 32 | Opt-out | Low | On | Off | Off | Internal | Proactive | On |
