# Supplementary material for: Key Information Influencing Patient Decision-Making About AI in Health Care: Survey Experiment Study
Source: J Med Internet Res. 2026 Jan 12;28:e75615. doi: 10.2196/75615 (PMC12795307; doi:10.2196/75615)
Supplement: Multimedia Appendix 3 [file jmir-v28-e75615-s003.docx]

Multimedia Appendix 3. Subgroup differences in effects of information factors on the probability of the AI device being trusted.

| Information Factors | Subgroups | AME^a^ (95% CI) |
| --- | --- | --- |
|  |  | Percentage Points |
| Added Value (Info present – info absent) |  |  |
|  | Need for cognition ^b^ |  |
|  | High (Mean + 1SD) | 16.21 (13.45, 18.97) |
|  | Moderate (Mean) | 14.27 (12.31, 16.22) |
|  | Low (Mean – 1SD) | 12.23 (9.47, 14.98) |
| HCP Oversight (Info present – info absent) |  |  |
|  | Familiarity with AI ^c^ |  |
|  | Somewhat/Slightly/Not at all | 19.27 (15.23, 23.32) |
|  | Extremely/Very | 11.30 (6.64, 15.95) |
|  | Last routine medical checkup ^d^ |  |
|  | Within last year | 18.27 (14.49, 22.04) |
|  | 1 or more years ago | 11.63 (6.25, 17.02) |
|  | Age group |  |
|  | 18-34 | 15.25 (10.43, 20.08) |
|  | 35-54 | 10.34 (5.15, 15.53) |
|  | 55 or older | 23.44 (17.78, 29.10) |
| Performance (High – Low) |  |  |
|  | Reading health literacy ^b^ |  |
|  | High | 22.02 (18.82, 25.22) |
|  | Low | 11.06 (7.85, 14.27) |
|  | Last routine medical checkup ^d^ |  |
|  | Within the last year | 20.09 (17.23, 22.95) |
|  | One or more years ago | 10.38 (6.32, 14.43) |
|  | Gender |  |
|  | Woman | 19.38 (16.10, 22.66) |
|  | Man | 12.85 (9.62, 16.08) |
| Regulatory Approval (Info present – info absent) |  |  |
|  | Reading health literacy ^b^ |  |
|  | High | 25.07 (20.25, 29.89) |
|  | Low | 13.63 (8.72, 18.53) |
|  | Last routine medical checkup ^d^ |  |
|  | Within the last year | 23.29 (18.98, 27.61) |
|  | One or more years ago | 12.92 (6.72, 19.12) |

a AME: average marginal effect. It is the average change in the predicted probabilities (percentage point increase or decrease) of the AI device being trusted/accepted across all participants when moving from one level of the information factor to the other, keeping all other variables in the model constant.

b Model adjusted for education level.

c Model adjusted for health literacy, numeracy, education level, and recency of last medical checkup.

d Model adjusted for health insurance coverage, age group, and perceived household financial status.
