# Supplementary material for: Key Information Influencing Patient Decision-Making About AI in Health Care: Survey Experiment Study
Source: J Med Internet Res. 2026 Jan 12;28:e75615. doi: 10.2196/75615 (PMC12795307; doi:10.2196/75615)
Supplement: Multimedia Appendix 4 [file jmir-v28-e75615-s004.docx]

Multimedia Appendix 4. Subgroup differences in effects of information factors on the probability of the AI device being accepted.

| Information Factors | Subgroups | AME^a^ (95% CI) |
| --- | --- | --- |
|  |  | Percentage Points |
| Added Value (Info present – Info absent) |  |  |
|  | Familiarity with AI ^b^ |  |
|  | Somewhat/Slightly/Not at all | 18.06 (15.11, 21.01) |
|  | Extremely/Very | 11.49 (7.95, 15.03) |
|  | Reading health literacy ^c^ |  |
|  | High | 19.34 (16.03, 22.65) |
|  | Low | 11.96 (8.64, 15.28) |
|  | Last routine medical checkup ^d^ |  |
|  | Within the last year | 19.19 (16.40, 21.97) |
|  | One or more years ago | 8.81 (4.76, 12.87) |
|  | Gender |  |
|  | Woman | 18.94 (15.54, 22.34) |
|  | Man | 11.38 (8.05, 14.71) |
|  | Race/Ethnicity |  |
|  | Person of Color | 19.64 (16.18, 23.09) |
|  | Non-Hispanic White | 12.01 (8.82, 15.19) |
| HCP Oversight (Info present – Info absent) |  |  |
|  | Familiarity with AI ^b^ |  |
|  | Somewhat/Slightly/Not at all | 25.34 (21.88, 28.8) |
|  | Extremely/Very | 9.59 (5.29, 13.89) |
|  | Last routine medical checkup ^d^ |  |
|  | Within last year | 21.92 (18.69, 25.15) |
|  | 1 or more years ago | 13.31 (8.33, 18.29) |
|  | Gender |  |
|  | Woman | 22.40 (18.07, 26.73) |
|  | Man | 14.01 (9.87, 18.16) |
|  | Age group |  |
|  | 18-34 | 13.77 (8.89, 18.65) |
|  | 35-54 | 13.05 (8.18, 17.93) |
|  | 55 or older | 30.13 (24.46, 35.79) |
| Performance (High – Low) |  |  |
|  | Familiarity with AI ^b^ |  |
|  | Somewhat/Slightly/Not at all | 18.18 (15.26, 21.09) |
|  | Extremely/Very | 10.58 (7.28, 13.88) |
|  | Reading health literacy ^c^ |  |
|  | High | 21.41 (18.22, 24.59) |
|  | Low | 7.66 (4.47, 10.86) |
|  | Numeracy ^e^ |  |
|  | High (Mean + 1SD) | 19.38 (16.22, 22.55) |
|  | Moderate (Mean) | 14.59 (12.34, 16.85) |
|  | Low (Mean – 1SD) | 9.77 (6.62, 12.93) |
|  | Last routine medical checkup ^d^ |  |
|  | Within the last year | 18.91 (16.29, 21.53) |
|  | One or more years ago | 7.03 (3.04, 11.02) |
|  | Gender |  |
|  | Woman | 18.71 (15.43, 21.98) |
|  | Man | 9.57 (6.37, 12.77) |
|  | Age group |  |
|  | 18-34 | 11.82 (8.14, 15.50) |
|  | 35-54 | 12.80 (9.01, 16.59) |
|  | 55 or older | 20.88 (16.53, 25.22) |
| Regulatory Approval (Info present – info absent) |  |  |
|  | Reading health literacy ^c^ |  |
|  | High | 19.93 (15.25, 24.62) |
|  | Low | 5.82 (0.90, 10.75) |
|  | Numeracy ^c^ |  |
|  | High (Mean + 1SD) | 15.79 (11.25, 20.34) |
|  | Moderate (Mean) | 12.93 (9.51, 16.35) |
|  | Low (Mean – 1SD) | 9.16 (4.18, 14.13) |
|  | Last routine medical checkup ^d^ |  |
|  | Within the last year | 18.65 (14.30, 23.01) |
|  | One or more years ago | 3.96 (-2.14, 10.06) |
|  | Gender |  |
|  | Woman | 16.58 (11.60, 21.57) |
|  | Man | 9.43 (4.58, 14.28) |

a AME: average marginal effect. It is the average change in the predicted probabilities (percentage point increase or decrease) of the AI device being trusted/accepted across all participants when moving from one level of the information factor to the other, keeping all other variables in the model constant. All analyses adjusted for other participant characteristics.

b Model adjusted for health literacy, numeracy, education level, and recency of last medical checkup.

c Model adjusted for education level.

d Model adjusted for health insurance coverage, age group, and perceived household financial status.
