# Supplementary material for: Key Information Influencing Patient Decision-Making About AI in Health Care: Survey Experiment Study
Source: J Med Internet Res. 2026 Jan 12;28:e75615. doi: 10.2196/75615 (PMC12795307; doi:10.2196/75615)
Supplement: Checklist 1 [file jmir-v28-e75615-s005.docx]

Checklist 1. CHERRIES (Checklist for Reporting Results of Internet E-Surveys) checklist.

| ***Item Category*** | ***Checklist Item*** | ***Explanation*** |
| --- | --- | --- |
| **Design** | Describe survey design | Inclusion criteria for this study include being an adult aged 18 or older, being proficient in reading and writing in English, and having had a primary care or cardiology care visit within the past three years. We oversampled racial and ethnic minority patients to ensure their representation was comparable to that of non-Hispanic white patients. This supported our ability to draw meaningful conclusions about diverse patient populations while accounting for historical underrepresentation in health research. |
| **IRB (Institutional Review Board) approval and informed consent process** | IRB approval | Review and approval for this study and all procedures were obtained from the Mayo Clinic Institutional Review Board (IRB # 21-012302). |
|  | Informed consent | The IRB granted a waiver of written documentation of informed consent. The first page of the survey displayed an IRB approved informed consent cover letter which provided information about the study, including its purpose, the investigator, the estimated length of the survey, data storage procedures, and contact information for the study team and the Mayo Clinic IRB. Participants were instructed to review this information and were informed that by proceeding to the survey, they were providing informed consent. |
|  | Data protection | No personal information was collected or stored with the survey responses. Study data were secured on institutionally approved and controlled access electronic storage. |
| **Development and pre-testing** | Development and testing | We conducted cognitive interviews (via Zoom, 30-45 minutes in length) with four participants to assess whether the survey questions were easy to understand and meaningful to participants. The interviewer shared the web-based survey with participants via shared screen and asked participants to verbally answer the survey questions and share their thought processes. The interviewer also asked participants to explain the information elements in their own words. The interviews were audio-recorded, and the interviewer took notes on reflections and observations. We revised the survey questionnaire based on interview notes. Following the cognitive interviews, we pilot tested the revised Qualtrics web-based survey with 30 participants to examine its feasibility and data quality. |
| **Recruitment process and description of the sample having access to the questionnaire** | Open survey versus closed survey | Closed survey. Only participants who consented to be part of the ResearchMatch platform and opted-in to be contacted and receive a link to our survey received an email invitation with a link to complete the online survey. |
|  | Contact mode | Participants received an IRB approved message describing our study via the ResearchMatch platform, where they could then decide to opt-in to be contacted and receive a link to our survey. ResearchMatch provided contact information (i.e., name and email address) for participants who opted in. Participants who opted-in received an email invitation with a link to complete the online survey. |
|  | Advertising the survey | ResearchMatch advertised our study via a contact message which is routed to potential volunteers who met study eligibility criteria. |
| **Survey administration** | Web/E-mail | The online survey was programmed and deployed using the Qualtrics survey platform. |
|  | Context | We recruited participants from ResearchMatch, a national health volunteer registry that was created by several academic institutions and supported by the U.S. National Institutes of Health as part of the Clinical Translational Science Award (CTSA) program. ResearchMatch has a large population of volunteers who have consented to be contacted by researchers about health studies for which they may be eligible. |
|  | Mandatory/voluntary | Voluntary survey. |
|  | Incentives | Respondents received a $10 gift card as remuneration for completing the survey. |
|  | Time/Date | August 2023 – September 2023 |
|  | Randomization of items or questionnaires | Display order of the two survey experiments were randomized for each participant. Display order of the AI prototypes within each experiment was randomized for each participant. |
|  | Adaptive questioning | N/A |
|  | Number of Items | Ranges from three to six questions per page. |
|  | Number of screens (pages) | 29 pages/screens in total including pages/screens with only instructions. |
|  | Completeness check | N/A. Participants can skip any question. |
|  | Review step | Back button was enabled to allow participants to review and change their answers. |
| **Response rates** | Unique site visitor | N/A |
|  | View rate (Ratio of unique survey visitors/unique site visitors) | N/A |
|  | Participation rate (Ratio of unique visitors who agreed to participate/unique first survey page visitors) | N/A |
|  | Completion rate (Ratio of users who finished the survey/users who agreed to participate) | N/A |
| **Preventing multiple entries from the same individual** | Cookies used | N/A |
|  | IP check | N/A |
|  | Log file analysis | N/A |
|  | Registration | Each eligible participants received a unique, single-use survey link in their email. Once that link has been used to complete the survey, the same link cannot be reused, which prevents multiple entries from the same individual. |
| **Analysis** | Handling of incomplete questionnaires | A total of 340 participants who completed at least 75% of the survey questions were included in the analysis. |
|  | Questionnaires submitted with an atypical timestamp | We did not exclude any responses based on time spent filling out the questionnaire. |
|  | Statistical correction | None used. |
